# Fungal Cell Factories for Efficient and Sustainable Production of Proteins and Peptides

**DOI:** 10.3390/microorganisms10040753

**Published:** 2022-03-30

**Authors:** Mette Lübeck, Peter Stephensen Lübeck

**Affiliations:** Department of Chemistry and Bioscience, Aalborg University, DK-9100 Aalborg, Denmark; psl@bio.aau.dk

**Keywords:** filamentous fungi, extracellular enzymes, myco-proteins, hydrophobins, anti-microbial peptides, production of recombinant proteins, precision fermentation, submerged fermentation, solid-state fermentation, agricultural side-streams

## Abstract

Filamentous fungi are a large and diverse taxonomically group of microorganisms found in all habitats worldwide. They grow as a network of cells called hyphae. Since filamentous fungi live in very diverse habitats, they produce different enzymes to degrade material for their living, for example hydrolytic enzymes to degrade various kinds of biomasses. Moreover, they produce defense proteins (antimicrobial peptides) and proteins for attaching surfaces (hydrophobins). Many of them are easy to cultivate in different known setups (submerged fermentation and solid-state fermentation) and their secretion of proteins and enzymes are often much larger than what is seen from yeast and bacteria. Therefore, filamentous fungi are in many industries the preferred production hosts of different proteins and enzymes. Edible fungi have traditionally been used as food, such as mushrooms or in fermented foods. New trends are to use edible fungi to produce myco-protein enriched foods. This review gives an overview of the different kinds of proteins, enzymes, and peptides produced by the most well-known fungi used as cell factories for different purposes and applications. Moreover, we describe some of the challenges that are important to consider when filamentous fungi are optimized as efficient cell factories.

## 1. Introduction

Filamentous fungi are excellent organisms as cell factories for production of a variety of products. They are robust and naturally produce efficient enzymes for the decomposition and conversion of biological material. They also produce different compounds, many of which can have interesting commercial applications. Filamentous fungi are present almost everywhere in all kinds of habitats, where their heterogenic lifestyle requires access to organic carbon. Fungi uptake inorganic material and from this, they can synthesize the biomolecules they need, including all amino acids. The inorganic material can be divided into macronutrients such as oxygen, hydrogen, nitrogen, phosphorus, potassium, sulfur, and magnesium, and micronutrients, i.e., manganese, iron, zinc, copper, and molybdenum, which are essential for fungal growth [1]. The organic carbon sources are derived from a large range of sources, ranging from single monomer sugars to complex polymers. Many fungi are saprophytes, where they play an important role in the environment as decomposers of dead organic material and as such are crucial for the conversion and mineralization of organic material [2]. They have developed an efficient biomass degradation apparatus and secrete plant cell wall degrading enzymes to retrieve nutrients from complex material [3,4,5,6]. Fungi have proven to be effective as industrial cell factories to produce a wide range of different products, due to their rapid growth, efficient utilization, and conversion of complex substrates into fermentable sugars. Among the most well-known ones are complex secondary metabolites such as antibiotics (e.g., penicillin), organic acids (e.g., citric acid) and various extracellular enzymes, including amylases and cellulases [7,8,9]. 

Filamentous fungi are thus getting increasing attention as workhorses in industrial production. Several recent reviews have focused on the use of fungal biotechnology in a bio-based circular economy, where novel discoveries and inventions open an amazing number of new opportunities for how to utilize fungi for the benefit of human life [10,11,12,13,14]. Hyde et al. [11] details 50 ways in which fungi potentially can be exploited. Among these prospects is an increasing focus on fungal production of proteins and food components thereby bypassing or supplementing animal production, which is associated with large agricultural land requirements and negative climate effects [10,15]. Animal production is expected to rise as the demand for animal derived products increases, not only due to the increasing population, but also due to the raise in income in many countries that previously could not afford animal-based food and had vegetal food such as rice, corn, and other cereals as staple food. A shift in the dietary pattern towards more sustainable food sources with better utilization of the arable land to sustain feeding the population without further burdening the environment is therefore highly relevant [15]. 

As cell factories, filamentous fungi play an increasing role in industrial production of proteins, especially enzymes, where more than fifty percent of all the industrial enzymes are produced by filamentous fungi [16]. Besides enzymes, other proteins such as specialty proteins and peptides with interesting functionalities, e.g., animal proteins for food, hydrophobins or anti-microbial peptides, is increasingly being developed for fungal production. The production can be carried out using native strains or engineered strains to produce multiple secreted proteins or to produce specific target proteins in highly specialized fungal hosts. Filamentous fungi offer certain advantages over other recombinant protein expression systems, which has become apparent over the years [16,17,18]. Among the arguments for using filamentous fungi as expression hosts for heterologous production of specific target proteins compared with bacterial or yeast hosts are their powerful secretory pathways, and their ability to perform various post-translational processing of eukaryotic proteins correctly. This includes glycosylation and disulfide bond formation like mammal cells [19]. 

This review focuses on (1) the use of filamentous fungi for production of proteins and peptides, especially of relevance for biomedicine, biomass deconstruction, feed, and food; (2) the challenges of culture conditions and fermentation techniques for efficient fungal production, and (3) the use of cheap substrates that are particularly relevant for the production of non-expensive biomolecules such as food proteins compared to the production of high-value products, e.g., pharmaceutical products. Down-stream product recovery post-fermentation is another challenge but will not be reviewed here, but instead we refer to [20,21,22].

## 2. Fungal Metabolism and Protein Secretion

Filamentous fungi naturally take up nutrients from the environment, which, depending on their lifestyle, can be dead plant and animal debris or from living organisms. Since they are heterotrophic, they are dependent on external carbon sources, but many of them are fully capable of absorbing inorganic nitrogen and other nutrients for their growth and metabolism. They can synthesize all the different amino acids needed from inorganic nitrogen. Fungal metabolism is usually divided into primary and secondary metabolism, and fungi can biosynthesize a large number of compounds with applications in various industries, most of which are excreted from the mycelium into the surroundings. In nature, many of these compounds are either used for nutrient recovery or are used in the organism’s battle with other organisms, e.g., secondary metabolites such as antibiotics. Compounds such as organic acids and enzymes can, besides being used for nutrient recovery, take part in the fight for nutrients with other organisms. The release of organic acid lowers the ambient pH to a level where many other organisms cannot thrive, and some enzymes aid in attacking and degrading living hosts, including other fungi (mycoparasites), plants (fungal plant pathogens), or insects (insect pathogens). 

When easily metabolized carbon sources, i.e., glucose, are present, the production of extracellular enzymes for degradation of complex biological material is inhibited, whereas the production of these enzymes is triggered by either starvation or by the presence of complex carbohydrates present in e.g., plant cell walls [23,24]. Effective degradation of plant complex carbohydrates requires a complex regulatory system to control the expression of such cell wall degrading enzymes. The production of extracellular enzymes is regulated at the transcriptional level, and several signal transduction pathways that control the expression of enzymes such as cellulases and xylanases have been identified [23,24,25]. Extensive analysis of the expression of enzymes and their promoters has provided great advances in understanding of the molecular mechanisms involved in cellulase and xylanase gene transcription regulation and is reviewed in [25]. However, many details in the precise signal transduction from sensing to regulation remain still largely unclear [25]. 

**Figure 1:**
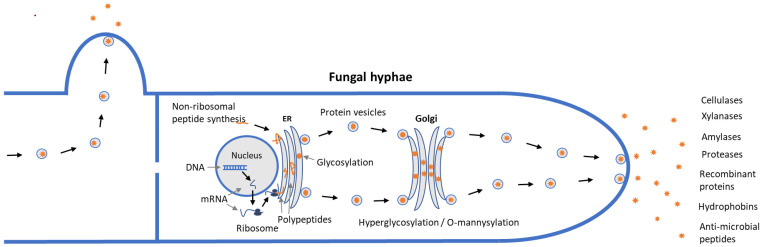
Fungal protein expression and protein secretion. Expression and secretion of proteins in filamentous fungi involve a set of important steps, starting with transcription of the protein encoding gene in the nucleus, transport of the mRNA to the ribosomes in the cytoplasm, polypeptide transfer from ribosomes to the endoplasmic reticulum (ER), protein folding and modification in ER, and further transfer of the folded proteins in vesicles to the Golgi apparatus, where they are further glycosylated and from there transferred through the outer membrane to the exterior. Peptides are produced by specific enzymes (non-ribosomal peptide synthases) in the cytoplasm and transferred to the exterior with the same protein secretion pathway as ribosomal produced proteins.

Production and secretion of proteins all the way from synthesis and transport to the cell’s exterior is a complex and highly regulated process in fungi [3,26] (Figure 1). Genes coding for extracellular proteins harbor a signal peptide part (15–36 amino acid long) in the N-terminal end. After gene transcription, the mRNA is translated to a polypeptide at the ribosomes in the cytosol. During the translation process, the ribosomes attach to the membrane of the endoplasmic reticulum (ER) by the help from a signal recognition particle (SRP), and the growing polypeptide is translocated into the lumen of ER. The signal peptide moiety is cleaved from the polypeptide by a signal peptidase upon entry into the ER lumen, and the resulting polypeptide is ready for folding and maturation. The polypeptide undergoes certain posttranslational modifications such as glycosylation followed by transport to the Golgi bodies, where further modification (O-mannosylation or hyperglycosylation) and folding takes place. The almost final proteins are escorted by cytoplasmic vesicles, transported to the outer membrane and then secreted out through fusion to the cytoplasm membrane (Figure 1). Highly secreted proteins contain efficient signal peptides, e.g., *T. reesei* cellobiohydrolase and *A. niger* glucoamylase, and even small differences between signal peptides can affect protein secretion [3,26,27]. 

As described, the secretory protein production in fungi is processed through ER, which is also the place where quality control of proteins is coordinated [28,29]. The folding of the polypeptide is facilitated by certain specific ER-resident proteins, e.g., foldases, chaperones and protein disulfide isomerases, and only correctly folded proteins pass through ER exit sites [3]. Glycosylation plays a role in protein folding, localization and stability and serves as information for the quality control in the ER. Protein glycosylation has two different variants: N- and *O*-glycosylation, respectively. The N-glycosylation of fungal proteins occurs first in the lumen of the ER while *O*-glycosylation occurs later in the Golgi apparatus [30]. Compared to yeasts, a distinctive feature of filamentous fungi is the polarized cell growth, where protein secretion is believed to occur primarily at the hyphal tip (Figure 1). Some fungi can be triggered to perform hyperbranching of hyphae and form larger amounts of hyphal tips, which appears to be beneficial for efficient protein secretion [31]. Morphology is thus believed to play a major role in efficient secretion of proteins [32].

## 3. Production of Native (Non-Recombinant) Proteins

### 3.1. Production of Enzymes for Plant Biomass Utilization

Fungi have adapted to very different environmental niches [33]. Their adaptation is eased by a diversity of fungal enzymes that are secreted extracellularly, and synergistically used for deconstructing various insoluble plant and insect polymeric substrates into soluble sugar nutrients. The capability of fungi to use solid substrates at low water content results in high concentration of secreted enzymes and high biocatalytic effeciency. The fungi access the solid plant materials by hyphal extensions and secrete the enzymes from the hyphal tips [34]. Fungi are of interest both as resources for the hunt for novel enzymes [35,36], as well as for production of enzymes and enzyme cocktails [5,37,38]. Among the enzymes are cellulases, amylases, pectinases, chitinases, proteases, and lipases that break down plant and insect biomass, e.g., cellulose, chitin, starch, pectin, proteins, and lipids [16,39,40,41,42,43]. Due to their versatility, a wide variety of fungi can be isolated from very different environmental niches, such as soil, compost, decaying wood, decaying plant material, building materials and different foodstuffs [33,35,36,42]. These niches include extreme temperatures and pH, although fungal growth is confined at temperatures beyond 65 °C [44,45]. From different genome projects, it is obvious that filamentous fungi in general contain a great variety of plant biomass-degrading enzymes in their genomes [8,46,47,48,49]. Furthermore, proteomic studies show that many of these enzymes are secreted when the fungi grow on lignocellulosic substrates [38,50,51,52,53]. The secreted part of proteins is called the secretome but the secretome obviously changes according to the circumstances under which the fungi grow such as cultivation conditions and availability of nutrients. The secretome includes liberally released proteins and proteins, attached to the outer cell wall [54]. Some fungi, especially within the genera *Trichoderma* and *Aspergillus*, which naturally secretes cellulases, amylases or other industrially relevant enzymes in large quantities, have been identified [55,56,57,58].

The application of plant biomass for production of various bioproducts in biorefineries using the biochemical route, involves pretreatment of the biomass to open the recalcitrant material and make it accessible for subsequent enzymatic hydrolysis to produce fermentative sugars for further processing [59,60]. The complete hydrolysis of lignocellulosic biomass requires an efficient cocktail of enzymes, which industrially most often are produced by filamentous fungi [38,52]. Still, none of those fungi can naturally secrete efficient cocktails containing all the necessary enzymes with highest activities in an optimal ratio. *Trichoderma reesei* is one of the most extensively used fungal species to produce cellulolytic enzymes in industry, due to its extraordinary high secretion capacity for enzymes, especially efficient cellulases [16,24,47,61,62]. Still, it lacks sufficient β-glucosidase activity for efficient cellulose hydrolysis and there are numerous examples on supplementation of cellulases from *T. reesei* with β-glucosidase preparations, especially from *Aspergillus niger* or other *Aspergillus* species [4,37,63,64,65,66]. However, industrial production of cellobiohydrolases and β-glucosidases in separate organisms is expensive because of the requirement for double equipment. This issue can be solved by co-cultivation strategies if *T. reesei* is cultivated together with *Aspergiilus* species [5,37,67] or by engineering filamentous fungi to produce multiple hydrolytic enzymes of desired top efficiencies in a single host, e.g., by introducing or up-regulating the demanded enzymes. The production of enzymes can be carried out on-site using cheap complex polymeric substrates e.g., containing agricultural sidestreams consisting of plant cell wall material for growth and enzyme production [38,68]. Co-cultivation with various fungi for enzyme production, especially using solid state fermentation (SSF) with different solid agricultural sidestreams, where the fungi do not necessarily have direct contact but can thrive in micro-niches in the substrate, has been shown in several cases to enable production of efficient enzyme cocktails [37,69], reviewed in [66]. These cocktails are likely optimized for hydrolysis of the same substrate that was used to produce them, as their secretomes may be induced specifically by the composition of the polymers present in the substrates. Further investigation of molecular interactions between fungi in co-cultures is necessary to fully understand them and to further optimize their production of enzyme cocktails [66].

### 3.2. Production of Mycoproteins for Food and Feed

Edible fungi have traditionally been part of the food system, especially mushrooms, where the fruiting body are used as food, or through food fermentation, including their widespread use in cheese production, e.g., blue, and white cheeses [70]. Fungal fermented non-animal foods are an integrated part of the diet in many countries, especially in East and South Asia and certain African regions. The fermented foods are divided into high-protein meat alternatives from legumes or cereals, such as Tempeh and Oncom, and into salty amino acid sauce and paste, e.g., shoyu and miso [70,71].

Mushrooms are the beyond ground fruiting bodies of macroscopic saprophytic fungi, most of which belong to the Basidiomycetes. Many of them are edible and have been used for food since ancient times, but only a smaller number are currently cultivated commercially [72,73]. The global mushroom market has grown considerably in recent years, and among the most important cultivated mushrooms are *Agaricus bisporus* (common mushroom), *Pleurotus ostreatus* (oyster mushroom), *Lentinula edodes* (shiitake), *Flammulina velutipes* (enoki mushroom), and *Volvariella volvacea* (paddy straw mushroom) [72,73]. Mushrooms are known for their umami taste and for their nutritional properties; they are low in fat, high in protein, and high in dietary fibers. In addition, they contain a range of vitamins (including vitamin B), minerals, antioxidants, and nutraceuticals [74,75,76]. Mushrooms are cultivated commercially on agricultural residues, enabling these waste materials to be converted into a valuable human food source [72]. They are traditionally eaten fresh but are increasingly used as dried powder (flour) to fortify various food types with nutrients and especially proteins as they contain up to 20–25% protein [73]. Such dried fungal products are called “mycoprotein”. As an example, *Pleurotus albidus* mycoprotein flour was used to replace wheat flour to produce cookies. The mycoprotein flour significantly increased the nutritional value of the cookies due to the contents of protein, dietary fiber, and phenolic compounds. Furthermore, the mycoprotein increased the hardness and altered the color of the cookies [77].

Besides these Basidiomycetes mushrooms, certain species of Ascomycetes and Zygomycetes microfungi have traditionally been used for food fermentation into meat-like products such as Tempeh (*Rhizopus oligosporus*) [78,79] and Oncom (*Neurospora* spp.) [80,81,82] in Asia. These microfungi have been found to contain higher protein in their mycelium than most Basidiomycetes mushrooms, and they are for this reason promising as alternative protein sources. For example, *Neurospora sitophila* mycelium contains 39–45% protein, 28–30% carbohydrates, 10–12% crude fats, 5% minerals and vitamins, and 3% fibers [80]. Traditionally, the base for production is SSF of particular soybeans but also other substrates such as chickpea, lupines, and various cereals are used [78]. A growing interest in these Asian food types are seen both in the US and in Europe, where several novel companies have started production of “Tempeh” style food types using local products, such as lupines and peas, especially using *R. oligosporus* (Table 1).

The production of mycoprotein using solid-state fermentation has recently been expanded to other substrates than the already eatable substrates, e.g., using low value sidestreams from the food industry such as brewers spent grain [81,83,84], pulp from sugar beets, potato starch production, coffee production or bran from flour production [85]. The protein content in many of these plant-based sidestreams is low, and they often exhibit low quality, poor digestibility, and a low-quality profile of amino acids, with a low amount of some of the essential amino acids, especially lysine, methionine, cysteine, and tryptophan. Some of the edible Ascomycetes fungi can break down plant proteins and non-digestible fibers, thereby making the fermented combined fungus-plant products higher in protein content, improve the amino acid profile, and increase the digestibility. They can provide new food properties, in terms of texture, flavor, and increased solubility [70,81]. In addition, fermentation can benefit the overall nutritional composition, as many of these fungi naturally synthesize vitamins such as B12, D6, and vitamin E, often lacking in other alternative protein products and in many of the plant-based sidestreams. Some of the plant-based sidestreams may contain anti-nutritional factors (ANFs), such as phytates and saponins, which may be reduced through the fermentation process, due to the action of phytases or other enzymatic activities. The ANFs can form insoluble complexes with valuable minerals such as Ca^2+^, Mg^2+^, Fe^2+^ and Zn^2+^, and thereby decreasing the bioavailability of the minerals. 

For these purposes, there is an interest for identifying new and better-adapted strains, and for starter cultures for fermentation of such sidestreams. These strains should possess a GRAS (generally regarded as safe) or Qualified Presumed of Safety (QPS) status [86,87], and if they do not, they will require an approval from EFSA for consumption in Europe. However, it is anticipated that there will be opportunities to expand the list of current strains with new strains, but the process of achieving GRAS or QPS status is a challenge.

Instead of the traditional use of microfungi for SSF of various food products (or solid sidestreams), there has been an increasingly interest to grow fungal mycelium in bioreactors, using submerged fermentation (SmF) in sugar-rich substrates [10,13,88]. An advantage of SmF is that fungi can assimilate inorganic N-sources and can synthesize all amino acids, thereby producing protein from protein-free feedstocks supplemented with an N-source, in contrast to other alternative protein sources, e.g., insects. Several startups have entered this area of producing mycelium-based food, especially using SmF (Table 1). The most well-known mycoprotein product on the market is called Quorn^TM^ (Table 1), which has been produced since 1985 [88]. Prior to commercialization, there has been 20 years of research and development, including a major screening process involving over 3000 fungal species [89]. *Fusarium venenatum* met all requirements of the screening, which included fast growth, filamentous morphology, lack of pigments, odors and toxins, and a protein content of over 45%. It was later approved for sale as a food protein source by the United Kingdom Ministry of Agriculture, Fisheries and Food [90], and is now sold in 17 countries [89]. Since the branched nature of fungal mycelium resembles muscle fibers, the mycoprotein can be used to achieve a meat-like texture in food products. Thus, the mycoprotein from *F. venenatum* is used as an ingredient in various products such as alternative chicken patties, sausages, and burgers. On a dry basis, *F. venenatum* can produce mycoprotein products with more than 60% protein [10]. 

Mycoproteins from *F. venenatum* and other mycelium fungi are promising sources of essential amino acids with an overall protein digestibility-corrected amino acid score of 0.996, indicating that they can be considered as high-quality protein [88]. Recent research has investigated the health benefits of mycoprotein products and found that they have a higher weight-percentage protein content than other common plant or fungal sources of protein. Furthermore, the fibers found in the cell walls of mycoprotein is comprised of two-thirds beta-glucan and one-third chitin, creating a “fibrous chitin–glucan matrix”, which is largely insoluble in the small intestine [88]. Review of 16 studies supports the role of mycoprotein in reducing overall cholesterol levels and short-term energy intake [88]. Compared to other protein food sources, mycoprotein products also contain reasonable amounts of vitamin B9 (folate), vitamin B12, calcium, phosphorous, magnesium, and zinc.

Besides their nutritional benefits, mycoproteins together with other microbial “single cell proteins” produced through fermentation can be good alternatives to support future food production. This is especially important for climate-friendly production since they do not require large area of land and the production has in general a low water consumption, in particular in the case of SSF. Furthermore, the production can be made independently of seasonal and climatic variations throughout the year, and with a lower greenhouse gas emission compared to plant protein sources [91].

## 4. Recombinant Production

Prior to progresses in genetic engineering, fungal strains were optimized through protoplast fusion, UV mutagenesis, etc., and selected, and the production capacity was optimized through fermentation technologies. Fungal protein production today is mainly based on engineered strains and improvements in fermentation technology for efficient production. This development may involve design and alteration of promoters, secretory signals and pathways, maturation of the nascent polypeptide in the endoplasmic reticulum (ER), hypersecretion stress and unfolded protein response (UPR), protein processing and modification in the Golgi and the subsequent secretion apparatus, reviewed in [3]. Fungal protein secretion is closely linked to cell and mycelial morphology, such as hyper-branching and tip density, which is related to culture conditions. For improving protein secretion, studies into hyphal compartments therefore may be useful [32].

Among the strongest arguments for using filamentous fungi as expression hosts for recombinant production of specific target proteins are their powerful secretory pathways, and their ability to perform various kinds of post-translational modifications of eukaryotic proteins correctly, including glycosylation and disulfide bond formation like mammalian cells [92,93]. Over time, it has become apparent that they offer certain clear advantages compared to other microbial protein expression systems, even though genetic manipulations are easier and quicker in the model organisms, *E. coli* and yeasts. Among advantages are that titers obtained for secreted proteins, surpassing 10–1000-fold what can be achieved with secretion using bacterial, yeast or mammalian cells. In these cells, protein secretion titers are often less than a few g/L [8]. Unlike expression platforms using bacterial (especially *E. coli*) or yeast (e.g., *Komagataella phaffii* (syn. *Pichia pastoris)* and *Saccharomyces cerevisiae*) [94,95,96], the development of filamentous fungal expression platforms is much more complex, time-consuming, and needs extensive development. Due to the great potentials and industrial interests, several reviews have addressed different aspects of recombinant production of proteins using filamentous fungi [3,26,93,97,98,99].

The most used filamentous fungi for recombinant protein production are *A. niger*, *A. oryzae*, *T. reesei*, and *Neurospora crassa* [7,56,57,100,101]. These fungi are quite well characterized by whole-genome-sequencing, transcriptomics, gene annotations, knock-out libraries, promoter libraries, known insertion sites, known signal-peptides, metabolic models, flux analysis and genetic engineering tools including adaptive laboratory evolution, and radiation [9,102,103,104]. Furthermore, several industrial filamentous fungi, e.g., *A. niger*, *A. oryzae*, and *T. reesei*, have been accepted as GRAS strains [105]. 

The recombinant production of proteins can be production of homologous proteins as well as of heterologous proteins, i.e., proteins from other organisms than the production host. Compared with heterologous proteins, homologous proteins are much more effectively expressed and secreted, as up to 1000 times higher production has been obtained [3,26]. Production levels of enzymes can reach tens of grams per liter in many fungal hosts [16], and genetically optimized hypersecreting strains of *T. reesei*, with cellulase titers of up to 100 g/L, have been established during the last decades [61,106]. According to Sun and Su [26], filamentous fungi account for approximately fifty percent of all industrial enzymes with production levels of 10–100 g/L. Therefore, there is a great interest in understanding the expression and secretion mechanisms of the host fungi. Recent research related to protein modification, maturation and secretion is reviewed by [27]. An advantage of using filamentous fungi for recombinant protein production is the relatively mature and cheap fermentation process, and that the extracellular proteins easily can be purified compared with intracellular proteins.

For most secreted proteins in filamentous fungi, post-translational modification by glycosylation plays an important role for protein folding, localization, stability, and for quality control before final protein maturation and secretion. Although yeast also can perform posttranslational modification, the glycosylation of filamentous fungi has been proposed to be superior [107]. However, the glycosylation pathways are very complex and still not fully understood. For this reason, engineering of glycosylation sites such as addition, deletion, or other modifications in the protein to be secreted appears to be a more efficient strategy for achieving improved protein secretion than modifying the glycosylation system of the fungal host [6,108].

For facilitating high transcription and improving mRNA stability of heterologous genes, it is important to achieve their integration at transcriptionally active regions of the host genome [3]. A strategy for obtaining a higher yield of recombinant proteins can be to disrupt genes encoding native high-yield proteins in the host to optimize the use of the energy reservoir of the cells [26]. Signal peptides are also very important for obtaining high yields of heterologous proteins [6]. A fungal host can secrete any protein (homologous or heterologous) provided that the protein has a signal peptide that is recognized by the host secretion pathway [26]. Homologous signal peptides are best recognized by the host, and small differences in the signal peptides can greatly affect secretion [6]. Heterologous fungal signal peptides are generally functional in other fungal hosts, though, whereas signal peptides from non-fungal organisms do not work [3]. Using signal peptides from the highly secreted proteins glucoamylase in *A. niger*, and the *T. reesei* cellobiohydrolase fused to heterologous proteins results in much better secretion in the respectively hosts. 

In addition, optimization of the nucleotide sequence of a gene of interest significantly can improve its expression level, thereby increasing the number of proteins entering the secretion pathway. The amino acid codons are degenerate, and the codon usage in the gene sequences can have large effects on mRNA stability and translation efficiency, which can result into varied amounts of protein [6]. 

Impaired heterologous protein production in filamentous fungi can often be related to proteolytic degradation, which causes low yields and a reduction in protein levels during processing [6]. The construction of protease-deficient strains of *Aspergillus*, *Trichoderma* and *Myceliophthora* sp. for application in heterologous expression of polypeptides, including cellulolytic enzymes, has been described [6,109,110,111]. Protease deficient strains have been obtained by mutagenesis and by direct gene knockout strategies [112]. Since fungal genomes contain a large number of proteases, strategies to enhance heterologous production have included deletion of the most secreted proteases or deletion of transcription factors that control expression of several extracellular protease-encoding genes. A transcription factor, *prtT*, was identified in *A. niger* and was found to have homologs in *A. oryzae* and *A. fumigatus* but was absent in *A. nidulans* [109,110,111]. Kamaruddin et al. [110] found that deletion of *prtT* increased the production of a heterologous protein approx. 35-fold in *A. niger*, and similarly deletion of the homolog in *A. fumigatus* decreased transcription of six genes encoding secreted proteases. Comparative secretomics is a powerful tool to efficiently identify target proteases and was used to identify and delete three target proteases in *T. reesei*. This resulted in a 6-fold increase in cellulase activity and a 78% decrease in protease activity [9].

Other attempts to increase the production of heterologous proteins have been overexpression of various ER proteins, e.g., chaperones, foldases, lectins, and nucleotide exchange factors. However, these efforts have not given clear results, as overexpression of one gene affected the expression of others, suggesting a complex regulation of the secretion pathway [26,30,113,114]. The molecular and physiological mechanisms of membrane traffic, i.e., secretory, and endocytic pathways, in *A. oryzae* and related filamentous fungi have been reviewed by Higuchi [115].

Most heterologous production of proteins is performed in standard hosts where genetic tools and experience are in place. Recombinant production may involve the use of the relatively novel gene editing tools including the CRISPR-based approaches, which have been established for a variety of filamentous fungi [116,117,118]. The selection of an appropriate host for the production of a specific protein is not easy to identify, as different strains and species may express certain proteins better than others. Recently, Jarczynska et al. [119] created a fungal gene expression platform (DIVERSIFY) that makes it possible to simultaneously express the same construct in different *Aspergillus* species for the purpose of identifying the best production host for the selected protein. The expression platform reduces the workload so that the construction of a single gene expression cassette can be used to transform all DIVERSIFY strains to identify the best host.

### 4.1. Production of Recombinant Enzymes for Plant Biomass Utilization

Agriculture primarily produces plants that can be used as food or as feed for livestock. Only part of the plants is used directly for food or feed (for mainly monogastric animals), and traditionally leave a large amount of plant biomass. Biorefining using these plant sidestreams is gaining increasing interest, to produce fuels, biochemicals or as nutrition for microbes (food or feed production). The sidestreams contain lignocellulosic plant cell walls, which pose significant technical and economic challenges, and require substantial pre-use processing. In most cases, biomass conversion is carried out in several steps: pretreatment, enzymatic hydrolysis of the plant polymers to monomeric sugars, followed by fermentation of monomeric sugars to the desired product, which finally must be recovered [5,120].

The enzymatic hydrolysis of plant biomass is performed using lignocellulosic enzymes, in particular cellulases. Efficient cellulolytic enzymes and other plant cell wall degrading enzymes are primarily produced by fungi, especially using *T. reesei* and various *Aspergillus* sp. as workhorses at an industrial level. These fungi and their enzyme systems have been central for many research programs, and the studies have greatly advanced the knowledge of production, secretion, and regulation of the relevant enzymes [3,9,26]. Since cocktails composed of quite a few enzymes are needed for efficient hydrolysis of plant material, several studies have focused on selection of microorganisms capable of secreting a high and diversified number of enzymes [35,36,40]. Studies have also focused on increasing the production efficiency of cellulolytic enzymes by optimizing the production and composition of the cellulolytic cocktail, including adding booster enzymes. Industrial strains of *T. reesei* and *Aspergillus* sp. have been developed into high performing cell factories for enzyme production, and the work has included trimming of the production hosts to produce the targeted (recombinant) products by optimizing and upregulating the enzymes [9,17,55,56,57,100,101,106]. 

Some of the specific challenges to produce endogenous enzymes are related to the tight regulation of glucose by the gene expression in filamentous fungi. In the presence of glucose in the fermentation medium, the expression of the endogenous enzymes is carbon catabolite repressed, which limits the production. There are two ways to overcome this, either by using inducing media, e.g., based on complex carbohydrates, or preferably to genetically modify the production strain by manipulating the carbon catabolite repression system, which in *Aspergillus* is encoded by CreA and in *Trichoderma* by Cre1 [93,106]. The famous *T. reesei* strain C-RUT30, derived from a large screening of mutants and selected for improved cellulase production, was later found to have deletions in the carbon catabolite regulator Cre1 gene [106]. 

Zhang et al. [93] review several strategies to increase the production of endogenous and recombinant lignocellulosic enzymes. These strategies include downregulation or deletion of genes involved in the PKA pathway, upregulation of the AMPK pathway, and overexpressing activators, which often have been found to be effective in increasing expression. At the same time, they point out that some of these changes may also cause deficiencies in strain growth and metabolism, and that a given strategy must be balanced between efficient protein production without impairing cell growth.

The industrial trimmed strains based on *T. reesei* and different *Aspergillus* sp. are also utilized as cell factories for production of a range of other relevant enzyme products, which are exploited by a broad range of industries, including food and feed, detergent, pulp and paper, and pharmaceutical [9,13]. 

### 4.2. Production of Animal-Derived Food Proteins 

Cellular food production (both non-GMO and GMO) has the potential to supplement or even substitute animal-based food production, due to decreasing costs and radically less climate impact compared to conventional egg, milk, and meat production [15,121,122]. Recombinant tools make it easier to manipulate genetic material from e.g., cattle in microbial cells to produce molecules with precise properties, commonly known as “precision fermentation”. The food industry can therefore in future design, produce and purify animal-derived proteins on a commercial scale [15]. Compared to plant proteins, the animal proteins generally have a higher nutritional value, and many of them, such as the whey proteins, are used as versatile ingredients in the food industry with many applications, due to their techno-functionalities as e.g., a gelling, foaming, or emulsifying agent. A major challenge in replacing animal products with animal proteins produced in microbes on a commercial scale is to ensure that the processes can be scaled up in a profitable way, as the food proteins have less commercial value compared to enzymes and pharmaceutical products in the fermentation industry. To keep production costs low, production could be carried out in biorefineries with lignocellulosic plant material, as shown by Wang et al. [123], who produced recombinant bovine and human αS1-casein using a hydrolysate from wheat straw lignocellulose.

Several different microbial hosts can be used for precision fermentation, and filamentous fungi are among the choices. When producing animal proteins in fungi, the challenges are related to similar issues as for expression of other recombinant proteins such as addition of a host signal peptide, optimization of the nucleotide sequence for the gene of interest, and that the production hosts can create glycosylation patterns that are different from the native protein. Thus, the recombinant proteins may differ in the physiochemical and functional properties [122].

The production of animal proteins in microbes, including filamentous fungi, is particular focused on milk and egg proteins. Among milk proteins, the whey protein b-lactoglobulin (BLG) is particularly interesting in this respect, as it is the main protein in whey. BLG has been shown to be an important component in many food products due to its versatile functional properties, as a gelling, foaming and emulsifying agent [122,124]. Moreover, non-denatured BLG has been identified to be able to modulate human immune response and increase human cell proliferation [125], which is important for human health. Other relevant milk proteins are the caseins, which are the most abundant protein components of milk (up to 80%) and are an important component of cheese. Additionally, caseins are used as food additives. Caseins are related phosphoproteins of different types, α-S1-, α-S2-, β-, and κ-casein, of which α-S1- and β-casein are the two most common caseins. Furthermore, β-casein comes in two genetic variants, A1 and A2, where A2 is preferable from a nutritional point of view, as the A1 variant has been linked to various chronic diseases [126], which although has not been confirmed by EFSA [127]. As BLG also is a key model protein in structural biology, it was recombinantly produced in yeast more than 20 years ago [128]. 

The field of expressing dairy proteins is rapidly increasing globally, and some small startups have already begun to commercialize animal free milk proteins to produce novel food products, e.g., Perfect Day with products on the market. From “first movers” such as universities, the field is expanding into leading global food companies, including the Finnish dairy company, Valio. The main drivers still seem to be the small startups, which are increasingly attracting amounts of venture capital. In addition to Perfect Day, some of the leading startups are: Change Food, ReMilk, FORMO, Those Vegan Cowboys, and New Culture. The Good Food Institute estimates that 50 companies are currently working on launching fermentation produced animal proteins. Several commercial companies already produce dairy proteins using yeast as their production platform, but the products may exhibit non-optimal post-translational modifications (PTMs). Keppler et al. [122] suggest using *E. coli* as a production host, and to produce the BLG intracellularly to obtain recombinant proteins without undesirable modifications. However, this requires troublesome extraction procedures from the cells. 

### 4.3. Production of Other Recombinant Proteins

#### 4.3.1. Production of Hydrophobins

**Figure 2:**
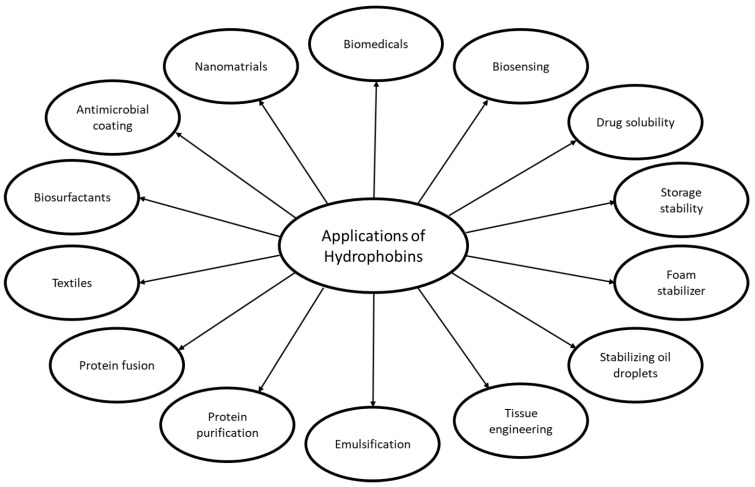
An overview of the industrial and commercial applications of hydrophobins.

Hydrophobins (HFBs) are low molecular weight (primarily less than 20 kDA) secreted proteins about 100 amino acids and are unique to the fungal kingdom except Yeast [129,130,131]. HFBs are found as multigene families with a low degree of sequence conservation, and they are characterized by the content of eight cysteine residues that form four disulfide bonds and contain 1α-helix and 2β-hairpins [132]. The HFBs form spontaneously amphipathic monolayers at hydrophobic-hydrophilic interfaces and are therefore important for the fungi in their biological cycle and their interference with the surrounding environment. Due to their surface-activity including biosurfactant and emulsifying properties, and antifoaming activity, they are considered to have many biotechnological applications [131,132,133] (Figure 2). The HFBs appear to be adapted to specific roles and are divided in 2 groups, Class I and Class II. Class I HFBs are less water soluble than Class II and have high variation among the proteins based on an inter-Cys-spacing construction, in contrast to HFBs from Class II, which are more conserved in sequence and inter-Cys-spacing [130,131,132]. Some HFBs appear to be intermediate and do not clearly belonging to one of the 2 classes [129]. Class I HFBs are involved in production of fibrillar structures (rodlets) that help fungal conidia bind to surfaces resulting in better resistance to the environment [130]. Class II HFBs have a role in signaling the moisture conditions on the spore surface, which has been described for *Trichoderma* [134]. If the water concentration decreases, the HFB concentration increases, which signals that the germination is not favorable, whereas the HFBs in wet conditions are low, which signals favorable germination conditions [134]. 

HFBs are produced in the cells, transported to the periplasm in lipid-enriched HFB vacuoles, and released to the exterior through the cell wall [134]. In connection to conidiation, HFBs are highly expressed so that HFBs can coat the spores, thereby supporting the attachment to different surfaces and protecting the fungi from stress conditions [134].

Another group of low molecular secreted proteins, the carbohydrate-binding proteins cerato-platanins (CPs), has been identified in various fungi [133]. Like HFBs, they are surface-active and share some functional and structural characteristics with HFBs, such as hydrophobicity, and that they form aggregate layers at hydrophobic/hydrophilic interfaces. CPs from some of these fungi show biosurfactant and emulsifying applicability. 

Due to the diversity and specificity of some of the HFBs, the variety of activities and lack of toxicity, there is a great interest in using HFBs for many different applications in the biotechnological, food, and pharmaceutical industry [131,135] (Figure 2). The proposed and tested applications include several drug-formulations including HFB fusion proteins, biosensor applications e.g., electrochemical biosensing, biomineralization, antimicrobial coatings, protein purification e.g., cellulases, immobilization of proteins and cells, fusion of anti-microbial peptides (AMPs) with HFBs for resistance to bacteria, foam stability in food for e.g., improved storage stability, emulsion of food products, gushing inducer in beer, stabilizing oil droplets, and many more are found [132,135,136,137,138]. Therefore, this has created an interest in development of cell factories in various organisms, including yeasts and filamentous fungi, for recombinant production of the different HFBs or engineered versions of the proteins [135]. Production yields are currently generally low and, therefore, optimization is needed to realize the industrial and commercial potential [131,135]. 

#### 4.3.2. Production of Anti-Microbial Peptides

Peptides are short biological molecules, less than 100 (mainly 2 to about 50) amino acids long and are found in all living organisms. In their active form, the peptides are either linear or cyclic, forming α-helices or β-sheets or combinations of both [139,140]. Fungi produce a vast number of peptides with different activities, of which the most well-known and studied peptides are those that exhibit cytotoxicity or antimicrobial functions (AMPs) [139]. Several studies in recent decades have shown that many of these harmful peptides exhibit antiviral, antibacterial, or antifungal functions, and they act as effective defense mechanisms for the fungi against other microbes in their environment [141]. Other peptides show cytotoxicity against nematodes, insects, or human cells such as cancer cells [142]. Although many studies have shown such activities, the molecular mechanism of the peptides and the induction of the genes expressing these peptides are often not or poorly known [140]. The peptides are encoded in the genomes, and many of them are not translated by the ribosomal system but are produced non-ribosomally [139]. The peptides can either be constitutively or expressed by induction [143]. The peptides contain a signal peptide like other secreted proteins and translocate to the ER for transport to the exterior [144]. Several studies indicate that peptides with cationic nature are targeting different ion channels or cell membrane components and suggested that they thereby disrupt the cell membrane e.g., by transmembrane pore formation [140,143]. In addition to the above functions, some of the peptides have been shown to possess other functions, such as e.g., the *Penicillium antifungal* (PAF) peptide involved in asexual development in *Penicillium chrysogenum* [145].

One group of peptides is CSαβ-type defensins, which are important components of the host’s immune system, but are not produced only by fungi [146,147]. Defensins are small, cationic, antimicrobial peptides 18–45 amino acids in lengths and most often contain 3 to 4 intramolecular disulfide bonds [146]. Eurocin, Micasin, and Plectasin are fungal defensin-like peptides (fDLPs) that are being considered for potential pharmaceutical applications [148,149,150].

Due to their antimicrobial and cytotoxic activities, there is a great deal of interest in developing these peptides into pharmaceutical agents [151] that can substitute microbial antibiotics and be used to treat serious diseases such as cancer [142]. Many of the peptides are not very effective as pharmaceuticals or show non-specific cytotoxity in their native form. For this reason, mutants are designed and developed by genetic engineering, which are then tested for better performance as medicine in treatment of various diseases [152]. An example is the fungal defensin Micasin from *Microsporum canis*, where a synthetically synthesized truncated version of the peptide binds the receptor-binding domain (RBD) of the COVID-19 spike protein six times better than the original mature peptide [153], potentially preventing or limiting the virus by penetrating into the cells. 

A challenge in producing these peptides in amounts for pharmaceutical purposes is that the peptides may be toxic to the expression host organisms. Many companies use their own preferred expression system and it can be expensive to choose another system. One way out of this may be to manipulate the preferred expression host. This was done in order to produce ETD151, a 44 amino acid long anti-fungal peptide (AFP), in an *A. oryzae* host, which does not tolerate EDT151, by altering the surface structure of the fungus [154]. Upon deletion of the potential binding domain on the surface, the fungus tolerated the peptides and was able to express EDT151 and several developed variants thereof.

## 5. Fermentation

Fungal cultivation and production can be carried out in two fundamentally different fermentation processes: (1) submerged fermentation (SmF) or (2) solid state fermentation (SSF) [155]. As the names suggest, the fungal cultivation is carried out in free-flowing liquid substrates in SmF, whereas the cultivation is carried out on solid substrates in SSF. Both processes are widely used for fungal cultivation and production [10]. SSF probably is the oldest technology, and is still used e.g., in Asia, for manufacture of alcoholic beverages such as *sake* or *koji*, and using soybeans for the manufacture of soy sauce, meat sauce, soybean paste, etc. [155]. In most cases, fermentation processes are performed under sterile conditions and with aeration and agitation, but some fermentations are carried out under unsterile conditions and without aeration, and agitation [156]. Today, many industries use SmF because of better control measures under these conditions than what is possible in SSF. For these reasons, SmF is the common method for most industrial fermentations, including commercial enzyme production [41,156]. Production results from SSF and SmF have shown that certain fungal strains perform better in SSF while other fungal strains perform better in SmF, when comparing the two processes. A choice of the fermentation process should therefore take into account the performance of the specific production strain into account, including the costs and benefits of each fermentation process, and select the most appropriate based on the selected strain [10,155]. The two fermentation processes are described in more detail below with examples on where each cultivation system is used. 

### 5.1. Submerged Fermentation

The fungal production in SmF is performed in liquid medium with free water and dissolved nutrients in controllable bioreactors. SmF can take place in three common ways: batch, fed-batch, and continuous fermentation. In batch fermentation, the medium is added and inoculated with the fungal strain at the start of fermentation, followed by a production period, after which the products are recovered. In fed-batch fermentation, the nutrients are added during fermentation, which can help increase cell density and production. In continuous fermentation, the nutrients are added during fermentation as in fed-batch, but at the same rate, the products are continuously recovered during the fermentation, which requires a steady state production with same amount of liquid flowing into the bioreactor as the amount flowing out [12,156]. 

In SmF, the various process operating parameters can be modified for the fungal strain and can be controlled continuously, such as temperature, pH, oxygen, mass transfer, and constant distribution of nutrients to fungal mycelia, resulting in high product yields [156]. However, the growth of the fungal strain during fermentation, especially in batch fermentation, changes the fluidity of the medium, which cause changes in the growth conditions, in particular the availability of nutrients and can also affect the availability of oxygen and pH. The varied changes in growth and conditions during the fermentation may affect the ability to secrete foreign protein [93,157]. For this reason, stains that are highly tolerant of stress are preferable [93].

There are several different bioreactor designs from simple without agitation to more advanced bioreactions with computer control and with very different volumes, from lab scale in microtiter plates [158] to commercial bioreactors with volumes over 300 m^3^ [156]. Among the advantages of SmF is that there is no limit to upscaling operations, and it is straight forward to control fermentation parameters such as pH, temperature, O_2_, CO_2_, mixing etc. Furthermore, it is possible to adjust the media composition during fermentation, and analyses can be carried out directly without any extraction process for extracellular products [156]. Today, SmF moves into Industry 4.0 using mathematical models of the processes, by accurate quantitatively describing the behavior of the culture and developing optimal model-based strategies for bioreactor operations [159].

For protein and peptide production in SmF, proteolytic degradation caused by the combined action of intracellular and extracellular proteases may occur during fungal growth, as previously mentioned [6]. If proteases are not deleted from the fungi as previously described, one way to avoid protease activity during fermentation is to add protease inhibitors, which may require specific inhibitors for different types of proteases. However, the addition of protease inhibitors may not be an economically viable solution on an industrial scale and may limit their use to small-scale protein expression [6]. 

There has been a great amount of work to investigate the influence of the morphology of the hyphae on the production and secretion of proteins. Since fungi are not single-celled but filamentous, and fungal hyphae in liquid culture grow heterogeneously, the macromorphologies range from dispersed hyphae, loose clumps to compact pellets, and the hyphae may be more or less branched. It is difficult to control the development of a specific macromorphology, as they are often unpredictable, which at the same time impacts the productivity of SmF [13].

### 5.2. Solid-State Fermentation

Fungal production in SSF is usually carried out on solid substrates surrounded by oxygen and with an appropriate moisture content to support fungal growth. Various solid substrates can be used such as grains, wheat bran, legumes, sugar beet pulp, spent grains, and other lignocellulosic plant materials. In Asia, SSF is used to make koji rice, which is rice fermented with *A. oryze*, as starters to produce sake and miso, or *A. oryze* fermented soybeans to make soy sauce, meat sauce, and soybean paste [155]. In these cases, *A. oryzae* is used to saccharify the grains or soybeans. SSF is also widely applied for mold-ripened cheeses and sausages [155], and as mentioned in an earlier section, to produce fermented mycoprotein-rich foods such as Tempeh or Oncom [78,79,80,81,82,155]. Like SmF, SSF can be used for production of proteins and peptides as well as for production of mycoproteins, although biomass harvesting from the fermentation residues is a major challenge as fungal hyphae are interwoven with the substrate [10]. SSF is particularly suitable for fermented mycoprotein-rich foods, where there is no problem with product recovery since the fermented solid substrate constitutes the product. 

SSF mimics the natural growth habitat of filamentous fungi, where during growth in SFF they secrete large amounts of enzymes and HFBs, which in many cases exceed the amount secreted in SmF [3,41,115]. However, understanding of genome-wide gene expression and the regulation of gene expression of lignocellulose degrading enzymes under SSF is limited compared to the knowledge generated from studies in SmF [3]. In *A. oryzae*, it has been found that certain proteins are secreted specifically in SSF, but not in SmF, e.g., the glucoamylase GlaB, whereas another Glucoamylase GlaA is produced in both SSF and SmF [115]. Thus, secretion of GlaB and GlaA seems to be regulated differently. Mixed cultures where two fungi are cultivated together is often also favored in SSF, as the fungi can take up different niches in the substrate, presumably withstanding any competition and thereby achieving stable interactions. Several examples have pointed to the prospects of producing enzyme cocktails using mixed fungal cultures to obtain a wider range of plant cell wall degrading enzymes compared to respective monocultures [5,37,115]. 

There are four bioreactor main types available for SSF: trays, packed beds, rotating (or stirred) drums, and forcefully aerated agitated bioreactors [41,160,161]. The selection of an appropriate bioreactor depends on the specific growth rate of the fungal strain and its tolerance to agitation, as e.g., trays do not facilitate agitation. The selection also depends on whether the production is a secreted protein or a fermented feed- or food product, as in the latter case there is no need for product recovery. Among the challenges in SSF is the control of process parameters, such as temperature, pH, moisture, and oxygen, which is more difficult than with SmF. This is due to the uneven distribution of fungal biomass, nutrients, moisture, temperature, and pH [10,41,160,161]. SSF has a much higher risk of bacterial contamination and problems with heat build-up, slower microbial growth, and product recovery. For SSF with agitation, there can be problems with slow continuous agitation and that continuous agitation has high-energy requirement [155].

According to Lee [155] and Manan and Webb [160], on the other hand, SSF has several advantages over SmF, as SSF exhibits higher volumetric productivity, uses less water and energy (especially if no agitation is required), generates less waste and is less time-consuming. In addition, it is a relatively inexpensive technology with the potential to use solid agro-industrial by-products or waste as substrates. Among the cases where it is particularly advantageous to use SSF is when the product has a solid form, consisting of the microbial biomass and residual solid substrate as is the case with fermented foods such as Tempeh and Oncom. Another case is if the product is only or primarily produced or produced more efficiently in SSF, which may be extracellular enzymes such as GlaB, or HFBs. Fungal conidia for inoculum can often be best produced in SSF, where they are more robust [162]. Finally, SSF uses solid residues, and there is a growing interest in exploiting agricultural, forestry and food processing residues in a sustainably way [161,163].

SSF is also difficult to scale-up due to possible contamination, low substrate utilization rate, and the lack of commercial SSF reactor designs [41,155,160]. Among the tools to facilitate the up-scaling of SSF bioreactors are to use mathematic models to integrate growth kinetics with process parameters such as energy, moisture, and oxygen consumption and to integrate monitoring devices into the bioreactors [160,161,164,165]. Among the main difficulties are the heterogeneity of the substrates and the fungal growth, which gives huge complexity on the micro-scale. 

## 6. Use of Biomass Streams as Fermentation Substrates

Fungal cell factories have a rapid growth rate on simple and inexpensive media in bioreactors. A well-designed growth medium is one of the key elements of a successful production, in addition to the choice of bioreactors and fermentation type. The medium components include C and N sources, minerals and water, and the design and optimization of fermentation media are crucial for the success of the fermentation processes [166]. Fungi as heterotrophs need organic carbon, but do not need organic N, since they are able to synthesize all the different amino acids needed for their growth and production as mentioned earlier. Different sources of N could be ammonia, ammonium salt, nitrate, or urea, and a proper carbon-to-nitrogen ratio should be present in the fermentation broth. A ratio of 10:1 is commonly applied to fungi for growth of biomass but can vary between different strains. In batch fermentation, a higher ratio will result in N depletion before all sugar is consumed, resulting in less efficient protein production [91]. For fungal cell factories that produce other products such as organic acids, N depletion is vital to stop growth and induce production [167]. 

In principle, sidestreams from various agricultural processes can be used as nutritious substrates for microbial production [168]. However, many sidestreams are solid and consist of lignocellulose, which require homogenizing, strong pre-treatment, and enzymatic hydrolysis, before they can be applied as hydrolysates for SmF [123]. However, plant juices from various industrial food production sources with readily available sugar content can be easily applied to SmF without heavy energy-intensive pre-treatments. Relevant plant juices include molasses, a by-product from sugar manufacturing, potato wastewater from the potato starch industry (potato juice) and brown juice, which is a residual juice containing glucose, fructose, amino-acids, and micronutrients from leaf protein production [91,168,169]. In order to be used as fermentation substrates, it is necessary to optimize the juices by adding missing components and in some cases, concentrating the nutrients. This can be performed through evaporation or through membrane-filtration, which is a feasible process, where initial microfiltration prior to nano-filtration ensures that the concentrated juice is at the same time sterile, since bacteria and fungal spores cannot pass through this membrane.

Solid sidestreams from local production of cereals, maize, oilseed rape, sugar and fodder beet, potato, grass, and legumes can be applied for SSF processes [81,84,85,163], instead of the harsh pre-treatment to produce readily available fermentable sugars for SmF [123]. Many of these sidestreams are currently underutilized or used as low-value feed or for biogas. There is a huge potential for upgrading these resources into novel uses, producing various innovative products in SSF using filamentous fungi. As the fungi secrete efficient lignocellulolytic enzymes, they are probably the best suitable microbes to utilize these side- and waste-streams. Traditionally, fermented mycoprotein-rich food is produced on foods [78,79,89], but there is an untapped potential in upgrading non-food substrates, e.g., spent grains, into novel mycoprotein-rich feed- or food ingredients [91,160].

## 7. Conclusions and Outlook

Filamentous fungi are efficient cell factories to produce proteins and peptides due to their efficient secretion systems. Several fungal strains have obtained GRAS status, and the experience gained includes fermentation upscaling expertise, relatively inexpensive growth, and production media, and improved molecular and genetic tools with the ability for post-transcriptional and post-translational modifications to construct relevant strains. Although filamentous fungi have many potential benefits in terms of recombinant protein and peptide production, research into their potential uses is in generally poorly funded and lags behind research in cell factories using bacteria and yeasts as production organisms. This has several reasons: The genetic tools are less developed compared to unicellular organisms [11,13]; the genomes of filamentous fungi, which are more complex organisms, contain far more genes, and the genome sequences of the first fungi were available much later than those of *S. cerevisiae* and *E. coli* and other bacteria [13]. Nonetheless, there has been a significant increase in knowledge about the various processes and molecular mechanisms involved in protein production and the secretory pathways in filamentous fungi in recent years. However, there is still a lack of basic understanding of how to obtain the full potential of fungal cell factories to produce heterologous proteins to the same extent as their own proteins.

One parameter that has only been covered to a minor extent in this review is the search for new fungal species and strains for the use as efficient future cell factories. As an example, we identified a new species in the genus *Aspergillus*, *A. saccharolyticus* [170], which has shown potential as a versatile and efficient cell factory [171,172]. It naturally produces β-glucosidases in large quantities and with higher enzyme efficiency than the well-known producer *A. niger* [171], and it has shown to complement *T. reesei* in co-cultures [37]. Furthermore, it is a good organic acid producer with a different acid repertoire than most *Aspergilli* [172], it produces only low amounts of secondary metabolites and no known mycotoxins [170]. It performs very well in bio-sidestreams [63] and has potential to achieve GRAS status for industrial production.

The potential for using edible filamentous fungi for the food system of the future, besides the well-known mushroom types, to upgrade agricultural sidestreams and to produce myco-proteins or other food proteins is expected to gain an increasing research focus and will provide a basis for novel food innovations. 

## Figures and Tables

**Table 1:** Examples of startup mycofood companies.

Company	Description
Beyond Coffee (DK)	Beyond Coffee collects coffee grounds and other types of biomass sidestreams to grow oyster mushrooms (fruit bodies), which are sold to restaurants. They sell mycelium and rent out ‘minifarm’ to canteens for harvest in the canteen. http://www.beyondcoffee.eu/ (accessed on 8 February 2022)
Contempehrary (DK)	Contempehrary produces and sales Nordic Tempeh (different types: fermented on oats, barley, rye, hemp, peas, or beans. Tempeh is made through SSF. https://contempehrary.com/ (accessed on 8 February 2022)
Enough Food (UK)	Enough Food produces fungal mycelium products using SmF and uses the trading name Abunda. They are a B2B company and expect to launch products in 2022. https://www.enough-food.com/ (accessed on 8 February 2022)
InnomyLabs	InnomyLabs works with the turn of mycelium into meat-analog products. They do not have products on the market. http://innomylabs.com/#!/-inicio/ (accessed on 8 February 2022)
Kernel MycoFood (USA)	Kernel MycoFood makes fungal food ingredients made by SmF of *Fusarium venenatum* (like quorn) https://www.kernel.bio/ (accessed on 8 February 2022)
Leep Foods	Leep Foods produces oyster mushrooms and blended products containing mushroom and meat. https://www.leepfoods.com/ (accessed on 8 February 2022)
Libre Foods (ES)	LibreFoods works with mycelium-based food products. Products not yet on the market. https://www.librefoods.co/ (accessed on 8 February 2022)
Meati (USA)	Meati produces whole cut mycelium-based products using SmF. They are in process with scaling their production. https://meati.com/ (accessed on 8 February 2022)
Mushlabs (DE)	Mushlabs uses fungi to up-cycle nutrients in sidestreams from agro- and food industries. Products not yet on the market. https://www.mushlabs.com/ (accessed on 8 February 2022)
Myco Foods (UK)	Myco Foods produces meat substitute products for the Food Industry https://www.mycofoods.co.uk/ (accessed on 8 February 2022)
MycoRena (S)	Mycorena produces Fungi-based alternative protein for the food industry using SmF. Promyc^®^ is a fungi-based natural ingredient to be used as meat replacement or dairy alternative. https://mycorena.com/ (accessed on 8 February 2022)
MycoTechnology (USA)	MycoTechnology makes mycoprotein-rich food ingredients based on fungal fermentation. https://www.mycoiq.com/ (accessed on 8 February 2022)
MyForest Foods (USA)	MyForestFoods is evolved from EcoVative, which produces various mycelium products. MyForestFoods have developed meat-free bacon. https://myforestfoods.com/home (accessed on 8 February 2022)
Mycovation (SGP)	Mycovation claims to be the first Asian start up to produce mycelium based food products. They do not have products on the market. https://www.mycovation.asia/ (accessed on 8 February 2022)
Tempty Foods (DK)	Tempty Foods is an early startup that produces Tempeh-like food products using SFF. They do not have products on the market yet. https://www.tempty-foods.com/ (accessed on 8 February 2022)
Quorn Foods (UK) *	Quorn Foods has been on the market for a long time. They produce and sell quorn and quorn products based on mycelium made by fermentation of *Fusarium venenatum* worldwide. https://www.quorn.co.uk/ (accessed on 8 February 2022)

* Well established and has been on the market for >20 years.

## Data Availability

Not applicable.
